# [Retracted] Naringenin has a chemoprotective effect in MDA‑MB‑231 breast cancer cells via inhibition of caspase‑3 and ‑9 activities

**DOI:** 10.3892/ol.2025.14923

**Published:** 2025-02-10

**Authors:** Rui Wang, Junhai Wang, Tianfu Dong, Jun Shen, Xitao Gao, Jun Zhou

Oncol Lett 17: 1217–1222, 2019; DOI: 10.3892/ol.2018.9704

Following the publication of this paper, it was drawn to the Editor's attention by a concerned reader that the DAPI-stained images featured in Fig. 3B on p. 1220 did not align properly with the anti-phospho-histone H3 (Anti-pH3) images shown in the adjacent column of data. Although the possibility of offering the authors an opportunity to publish a corrigendum was considered, an independent review of these data in the Editorial Office revealed that the images included in this figure had appeared previously in different form in a different paper published by different authors at different research institutes in the journal *PLoS Biology.* Owing to the fact that the contentious data in the above article had already been published prior to its submission to *Oncology Letters*, the Editor has decided that this paper should be retracted from the Journal. The authors were asked for an explanation to account for these concerns, but the Editorial Office did not receive a satisfactory reply. The Editor apologizes to the readership for any inconvenience caused.

